# ANALYSIS OF CONVERSATIONAL ENTRAINMENT AMONG OLDER PERSONS WITH ADRD AND UNIMPAIRED ADULTS

**DOI:** 10.1093/geroni/igad104.2742

**Published:** 2023-12-21

**Authors:** Christine Williams, Joseph McKinley, Charles Cooper, Nurgun Erdol, Christopher Beetle

**Affiliations:** Florida Atlantic University, Boca Raton, Florida, United States; Florida Atlantic University, Boca Raton, Florida, United States; Florida Atlantic University, Boca Raton, Florida, United States; Florida Atlantic University, Boca Raton, Florida, United States; Florida Atlantic university, Boca Raton, Florida, United States

## Abstract

Social isolation threatens the health of older adults, particularly those with Alzheimer’s disease and related dementias (ADRD). Our research uses measures of behavioral coordination to quantify the entrainment of an individual’s behavior to the collective dynamics of a group to which they belong, aiming to identify useful strategies to reduce social isolation for persons with ADRD in therapeutic group contexts. The present study is based on data collected during 27, 30-minute group therapy sessions conducted biweekly via WebEx by staff facilitators at a university-affiliated Memory and Wellness Center (MWC) in 2021, when CoViD-19 restrictions limited patients’ access to existing, in-person day programs. We study conversational entrainment among facilitators and participants in the recorded sessions using previously identified measures such as speech duration; voiced and voiceless interval durations; variations in pitch, intensity, and syllabic rate; and turn breaks. We present preliminary results identifying dynamical characteristics of sessions showing greater and lesser degrees of group social engagement before discussing possible strategies facilitators could use to enhance social coordination within therapy groups. The mental health benefits of professionally facilitated supportive group discussions for older adults with ADRD are well-accepted. Measuring conversational entrainment has the potential to provide quantitative evidence of cognitive benefits for persons with ADRD, and to help shape a therapeutic environment tailored to the needs of this vulnerable population.

